# Divided by choice? For‐profit providers, patient choice and mechanisms of patient sorting in the English National Health Service

**DOI:** 10.1002/hec.4223

**Published:** 2021-02-05

**Authors:** Walter Beckert, Elaine Kelly

**Affiliations:** ^1^ Department of Economics, Mathematics and Statistics Birkbeck University of London London UK; ^2^ Institute for Fiscal Studies London UK

**Keywords:** contracting out public services, demand for healthcare, inequality, patient choice

## Abstract

This paper studies patient choice of provider following government reforms in the 2000s, which allowed for‐profit surgical centers to compete with existing public National Health Service (NHS) hospitals in England. For‐profit providers offer significant benefits, notably shorter waiting times. We estimate the extent to which different types of patients benefit from the reforms, and we investigate mechanisms that cause differential benefits. Our counterfactual simulations show that, in terms of the value of access, entry of for‐profit providers benefitted the richest patients twice as much as the poorest, and white patients six times as much as ethnic minority patients. Half of these differences is explained by healthcare geography and patient health, while primary care referral practice plays a lesser, though non‐negligible role. We also show that, with capitated reimbursement, different compositions of patient risks between for‐profit surgical centers and existing public hospitals put public hospitals at a competitive disadvantage.

## 1 INTRODUCTION

Many government‐funded services are outsourced to the for‐profit sector (European Commission, 2015; Mossialos & Wenzl, 2016). Use of for‐profit providers in government‐funded healthcare has been introduced in the USA: Medicare covers procedures in ambulatory surgical centers (ASCs) since 2008; the Tricare Prime program provides military health insurance via a network of supplemental for‐profit contractors, and the Department of Veterans Affairs considers a similar scheme. In the UK, a series of policy reforms in the 2000s allowed for‐profit providers to treat patients funded by the National Health Service (NHS; Anell, 2015; Bergman, Johansson, Lundberg, & Spagnolo, 2016; Cooper, Gibbons, & Skellern, 2018). Such restructuring typically aims at expanding capacity, reducing waiting times, enhancing patient choice, and thereby intensifying competitive pressure on incumbents, to improve efficiency, quality, and innovation.

Assessments of the impact of such reforms primarily focus on their success of delivering aggregate quality or efficiency improvements that benefit all (Besley & Malcomson, 2018; Bergman et al., 2016; Cooper et al., 2018). However, such reforms are often accompanied by concerns about the way such benefits are distributed across different types of patients. In particular, there is a concern that the new providers will exacerbate existing inequalities by location, socioeconomic status, ethnicity, or underlying health. While debates are often sharpest in publicly funded healthcare systems like the NHS, similar sentiments have been expressed in the United States, for example by Suskind et al. (2015) with regard to ASCs. While such concerns are predominantly driven by equity considerations, inequalities in the use of, and access to, private providers may also have important implications for the competitive pressures and costs faced by public hospitals (Pérotin, Zamora, Reeves, Bartlett, & Allen, 2013; Meinow, Parker, & Thorslund, 2011) or for the reallocation of funds.^1^

In this paper, we investigate the distributional consequences of the NHS reforms. Equity concerns are particularly acute in a publicly funded healthcare system that aims to provide equal access for equal need. We ask whether any gains resulting from the expansion of access to for‐profit providers benefit all patient equally. We also explore what mechanisms cause inequality in access. In counterfactual simulations, we explore the differences in benefits between wealthy/poor and ethnic majority/minority patients but for differences in health geography, primary care (GP) referral practice, patient health, and patient preferences. Furthermore, we ask whether any adverse consequences for the allocation of costs between public and for‐profit providers undermine a level playing field between providers.

We pursue these questions within the context of structural reforms in the English NHS allowing for‐profit hospitals (Independent Sector Providers, ISPs) to enter the market for specific publicly funded elective procedures. ISPs arguably provide shorter waiting times, higher patient satisfaction, lower hospital‐acquired infection rates, and higher clinical quality (Browne et al., 2008; Care Quality Commission, 2012). By 2012/2013, ISPs treated 20% of NHS‐funded patients. ISPs disproportionately treat wealthier and healthier patients (Bardsley & Dixon, 2011; Chard, Kuczawski, Black, & van der Meulen, 2011). Patients in the top quintile of the wealth distribution are 85% more likely to be treated at ISPs than those in bottom quintile. Ethnic majority patients are more than 2.5 times as likely to be treated at ISPs than ethnic minority patients. At the same time, by 2012/2013 all providers are paid according to national tariffs. In this study, we consider NHS‐funded hip replacement patients in 2012/2013.

Who benefits? Our analysis shows that patients in the top three quintiles of the wealth distribution benefit twice (thrice) as much as those in bottom fourth (fifth) quintile; and experience a reduction in inequality in choice opportunities, while the two bottom quintiles do not. White patients benefit six times as much as ethnic minority patients; and experience an attenuation in inequality of choice opportunities; ethnic minority patients do not. What explains differential benefits? Our counterfactuals reveal that about two thirds of the difference between rich and poor patients are due to local health geography, and one fifth due to patient health and GP referral practice; and about half of the difference between ethnic majority and minority patients are due to local health geography and patient health. What is the extent of externalities due to differential access? We find that sorting of less complicated hip patients into ISPs imposes costs of ca. £0.5m p.a. on public hospitals.

Our work contributes to three main literatures. First, our demand analysis contributes to the hospital choice literature (Gutacker, Siciliani, Moscelli, & Gravelle, 2016; Ho, 2006; Beckert, Christensen, & Collyer, 2012; Gaynor, Propper, & Seiler, 2016; Capps, Dranove, & Satterthwaite, 2003; Kessler & McClellan, 2000; Moscelli, Siciliani, Gutacker, & Gravelle, 2016; Sivey, 2012). As Gutacker et al. (2016) and Moscelli et al. (2016), we incorporate the introduction of for‐profit providers, which are not included in many other analyses of hospital choice in England (Gaynor et al., 2016)^2^. We expand this literature by using our structural approach in counterfactual simulations to establish causal effects.

Second, we add to a small literature on the impact of new and specialized providers on the market for elective health care. This literature includes for‐profit providers in publicly funded health systems (e.g., Bardsley and Dixon, 2011; Chard et al., 2011; Cooper et al., 2018) and ASCs in the US system (Courtemanche & Plotzke, 2010; Munnich & Parente, 2018; Plotzke & Courtemanche, 2011; Suskind et al., 2015). This literature has primarily highlighted efficiency benefits following entry (Cooper et al., 2018). Our contribution is to show how and through what channels any benefits of for‐profit provision are distributed.

Finally, we add to the literature on socioeconomic inequalities and inequities in health care utilization. There exists descriptive work on differences in service use and outcomes (Doorslaer, Koolman, & Jones, 2004; Morris, Sutton, & Gravelle, 2005; O'Donnell & Propper, 1991; Cookson, Laudicella, & Donni, 2012) and on variation in the quality and types of care received by different types of patients (Fiscella, Franks, Gold, & Clancy, 2000; Moscelli, Siciliani, Gutacker, & Cookson, 2015). But this descriptive literature remains speculative about likely pathways through which socioeconomic position and race affect health care.^3^ Our counterfactuals go further and permit an analysis of the mechanisms through which inequalities and inequities in health care come about.

Our results have policy implications. They contribute to the evidence based on ethnic disparities in healthcare access (Fiscella et al., 2000; Cookson, Propper, Asaria, & Raine, 2016; Dixon, Robertson, Appleby, Burge, & Devlin, 2010; Nelson, 2002) in a system that operates under the mandate of providing equal access for equal needs. They also lend support to calls for more NHS support of poor and minority groups (Pinder, Ferguson, & Moller, 2016) and for continued monitoring more generally (Exworthy, Blane, & Marmot, 2003).

We also contribute to ongoing debates in many jurisdictions on the role of for‐profit providers in publicly funded health and social care. Our results demonstrate that the location of providers has important implications for how benefits to patients are distributed.^4^ They caution that where reforms create brand new entrants there may be unintended consequences that some parts of the population are better served than others (Anell, 2015); this has also been observed for ASCs in the United States (Suskind et al., 2015).

More generally, our results suggest that there are gains from opening up public services like health care and education to for‐profit provider competition, but these gains may accrue to those service users who can make choice‐based systems work for them. This may therefore influence policy design whenever governments, federal agencies and public bodies have equity objectives.

The rest of the paper is organized as follows. Section 2 describes the empirical setting of our study. Section 3 outlines our econometric approach, and Section 4 summarizes our headline estimates. Section 5 provides counterfactuals simulations, and Section 6 concludes.

## 2 BACKGROUND AND DATA

NHS reforms 2003–2008 allowed two types of for‐profit providers (ISPs) to treat NHS‐funded patients. The first type are Independent Sector Treatment Centers (ISTCs). These are private facilities under contract with the NHS that are awarded with a view to locally alleviate capacity constraints for routine procedures.^5^ Most ISTC contracts had expired by 2012/2013, the period we study. The second type are conventional private hospitals that from 2008 are allowed to compete for NHS‐funded patients, with a view to enhance choice and competition. ISTCs' locations are a policy response, private hospitals' locations are not. It is important to recognize, however, that private hospitals are typically located on sites of former public hospitals, notably in urban, densely populated areas, and therefore largely predetermined.^6^ Patients' eligibility of access to both is subject to restrictions based on underlying health, primarily because ISTCs and many private hospitals do not have intensive care facilities.^7^ By 2012/2013, payments for ISPs were based on national tariffs for Health Resource Groups (HRGs).^8^

We use data from the 2012/2013 wave of Health Episode Statistics (HES). These are administrative records of NHS‐funded inpatient treatments in England. Our sample consists of 62,695 hip replacement patients. They were treated at one of 195 public hospitals or 119 ISPs. Table 1 provides a summary of patient characteristics by provider type. The share of ethnic minority patients is much lower among ISP patients (1.3%) than among NHS patients (3.9%)^9^. Almost 20% of white patients were treated by ISPs, compared to just 7.5% of nonwhite patients. This is consistent with qualitative evidence on how patient choice operated during this period, where GPs voiced concerns that language barriers may limit the ability of ethnic minority populations from exercising choice (Dixon et al., 2010).

**TABLE 1:** Mean patient characteristics by chosen provider type

	ISP	NHS	Difference
Age	68.2	68.6	−0.4***
	(10.0)	(11.6)	(0.1)
Ethnic minority	0.013	0.039	−0.025***
	(0.115)	(0.193)	(0.002)
Female	0.598	0.601	−0.002
	(0.49)	(0.49)	(0.005)
Local area deprivation	0.391	0.45	−0.058***
(Scaled 0–1)	(0.253)	(0.275)	(0.003)
Moderate comorbidity	0.167	0.225	−0.058***
(CI = 1)	(0.373)	(0.418)	(0.004)
Severe comorbidity	0.044	0.096	−0.052***
(CI > 1)	(0.205)	(0.295)	(0.003)
Prev emergency admission	0.132	0.230	−0.098***
	(0.338)	(0.421)	(0.004)
Prev elective admission	0.481	0.568	−0.088***
	(0.500)	(0.495)	(0.005)
GP ref Herfindahl–Hirschman Index (2011)	0.548	0.607	−0.059***
	(0.178)	(0.197)	(0.002)
GP 3 years ISP ref share	0.13	0.077	0.054***
(2009/2010–2011/2012)	(0.106)	(0.086)	(0.001)
N	12,357	50,525	

*Notes*: Local area deprivation is measured using the (inverse) rank of the patient's Lower Super‐Output Area's Index of Multiple Deprivation in 2001. This measure is then rescaled to fall between zero and one. Ethnic minority are those not classed as White British or Irish. Comorbidities measured using the Charlson Index calculated using the information in the hip replacement admission. Previous admissions in the previous 3 years (1095 days) for any cause. Sample includes patients that had an elective hip replacement in 2012/2013 and were treated by a hospital that treated at least 19 other patients and that was among the closest 10 providers from the centroid of the patient's LSOA or a specialist hospital within 50 km.

Abbreviations: ISP, Independent Sector Providers; LSOA, Lower Super Output Area; NHS, National Health Service.

As measures of patient health, we use the Charlson Index of comorbidities^10^ and indicators for whether the patient had at least one (NHS‐funded) elective or emergency admission in the three years (1095 days) prior to the hip replacement admission, for any cause. Our measures confirm that ISP patients are on average less complex and have better underlying health than NHS hospital patients.^11^ It is however important to note that the market is not completely segmented by underlying health: a substantial fraction of ISP patients do have comorbidities or prior admissions.^12^

We embed characteristics at the neighborhood level via the patient's postcode district and LSOA.^13^ Socioeconomic status is measured using the neighborhood level Index of Multiple Deprivation (IMD) as compiled by the Office for National Statistics.^14^ This measure allows us to rank neighborhoods from the least to the most deprived. We rescale the IMD, henceforth referred to as “deprivation,” to lie between zero and one. Higher values imply higher deprivation. As documented by Chard et al. (2011) and elsewhere, ISP patients are on average less deprived than patients that are treated by NHS hospitals. In our sample, the average NHS patient lived in an area with a deprivation rank of 0.45, compared to 0.39 for the average ISP patient. At the patient level, the share of patients treated by ISPs in the top three, richest quintiles exceeds 20%. This falls to 16% in the fourth quintile and to 12% in the fifth quintile of the deprivation distribution.

HES permit quantifying historic referral patterns of the patient's GP. From HES outpatient records detailing GP practice referrals in 2011/2012 in the Orthopedics and Trauma specialty, we calculate a Herfindahl–Hirschman Index (HHI) of the concentration of referrals across providers for each GP practice.^15^ We also use all referrals from 2009/2010 to 2011/2012 to calculate the share of referrals to ISPs over those three years. Table 1 shows that patients who choose ISPs are registered at GP practices with lower concentrations of referrals. The average patient treated by an ISP was registered with a GP practice that referred 13.2% of patients to ISPs, compared to an average of 7.6% for those treated by an NHS hospital.

Table 2 provides a summary of hospital attributes, by type of provider. The hospitals in our sample conducted at least 20 NHS‐funded hip replacements in 2012/2013. Of these, 119 (or 38%) are ISPs. This share is higher than the share of patients treated by ISPs of just over 20%, because ISPs treat fewer patients per hospital (103 on average, compared to an average of 253 for NHS hospitals). While a large range of quality measures is recorded for NHS hospitals, very few of these are available for ISPs. All the quality measures we use are therefore constructed using the information available in HES.^16^ We proxy for hospitals' clinical quality using the ratio of observed 30‐day all‐cause emergency readmissions for hip replacement relative to expected readmissions at the hospital level, given the hospital's case mix. Readmissions include any emergency readmission to any hospital for any cause within 30 days. Expected admissions at the hospital level are constructed by regressing readmissions on age, sex, and prior admissions, and underlying comorbidities of hospital patients. A ratio of unity indicates that the rate of readmissions is as expected, higher ratios imply lower clinical quality. The mean readmission ratio is higher for NHS hospitals than ISPs. This corroborates existing descriptive evidence of on average higher clinical quality at for‐profit hospitals (Browne et al., 2008; Care Quality Commission, 2012; NHS Partners Network, 2015).^17^ The table also shows that the average waiting time at a public hospital is about twice that at an ISP.

**TABLE 2:** Means of hospital attributes, by provider type

	NHS	ISP	All
Attributes with RC			
30 Day Em Readmit Ratio (2012)	0.91	1.09	1.02
	(0.42)	(0.64)	(0.53)
Attributes without RC			
Median waiting time	87	45.3	71.2
	(23.3)	(33.2)	(34.1)
Share early Foundation Trusts	0.160	N/A	0.10
	(0.37)	N/A	(0.30)
Share specialist hospitals	0.0205	0.008	0.016
	(0.142)	(0.091)	(0.125)
Patients	253.3	103.4	196.5
	(174.0)	(96.0)	(166.0)
Hospitals	195	119	314

Notes: Median waiting times for 2012/13, in days, are measured from the date of the decision to admit for a procedure and to the date of the admission for the procedure.

Abbreviations: ISP, Independent Sector Providers; NHS, National Health Service; RC, random coefficient.

## 3 ECONOMETRIC MODEL

We use a random utility model (RUM) to describe the patient's discrete hospital choice problem. We consider a mixed multinomial logit (MMNL) model that allows us to capture patient level heterogeneity and exhibits flexible substitution patterns without imposing a correlation structure across choice alternatives.

More tightly specified alternatives in the logit family, for example, conditional or nested logit, while yielding more efficient estimates, risk being misspecified and consequently inducing inconsistent estimators. As demonstrated by McFadden and Train (2000), an appropriately rich MMNL specification can arbitrarily closely approximate any RUM for discrete choice. This flexibility renders it an attractive econometric framework for analysis. Incidentally, Gaynor et al. (2016) inspired our modeling approach, suggesting that the patient choice model could be estimated by a random coefficients logit (or MMNL) model.^18^

At the same time, our model specification is relatively parsimonious, including only those alternative specific attributes and patient characteristics that have been shown to be most salient, because the model is intended as a tool for counterfactual simulations. Richer specifications would reduce estimation bias, but at the cost of larger variance and reduced predication accuracy; this trade‐off is discussed in the statistics literature (Breiman, 1995; Tibshirani, 1996).

Consider hip replacement patient *i*. Let *g*(*i*) denote *i*'s GP (practice).^19^ And suppose that *g*(*i*) offers *i* to choose among a set of NHS hospitals $N_{g\left(i\right)}$ and a set of ISPs $I_{g\left(i\right)}$. Then, patient *i*'s choice set is given by $J_{g\left(i\right)}=N_{g\left(i\right)}\cup I_{g\left(i\right)}$. Let *U*_ij_ denote *i*'s indirect conditional utility from having the procedure carried out at hospital *j*, $j\in J_{g\left(i\right)}$, and consider the specification

$$U_{ij}=x_{ij}^{\prime }\beta_{i}+\epsilon_{ij},$$

where ***x***_ij_ is a K‐vector of hospital attributes that may vary across patients. The attributes we consider are distance between patient Lower Super Output Area (LSOA) centroid and hospital site, hospital quality as measured by the ratio of observed to expected 30‐day readmissions and by a Foundation Trust dummy, waiting time, and dummy indicators for ISPs and specialist orthopedic hospitals. We also include pairwise interactions between the hospital attributes and hospital fixed effects.^20^

The vector *β*_*i*_ is a vector of possibly random coefficients,

$$\beta_{ik}=\beta_{k}+z_{i}^{\prime }\theta_{k}+\sigma_{k}\nu_{ik},\;k=1,\ldots ,K,$$

where ***z***_*i*_ is a vector of patient level characteristics, *σ*_ik_ > 0 for random coefficient and zero otherwise, and *ν*_ik_ is an independent standard normally distributed random variable. The patient characteristics we consider are patient age, local area deprivation of the patient's LSOA, the referral HHI and referral share to ISPs over the last three years of the patient's GP, and dummy indicators for ethnic minority status, gender, for the presence of moderate and severe comorbidities, and of previous emergency admissions and elective admissions, respectively.

In this model, *β*_*k*_ + ***z***_*i*_′*θ*_*k*_ captures the conditional mean of the random coefficient *β*_ik_ on hospital attribute *k*, given patient characteristics ***z***_*i*_, or the observed heterogeneity in *i*'s valuation of attribute *k*. The contribution *σ*_*k*_ *ν*_ik_ to *β*_ik_, in turn, captures unobserved heterogeneity in *i*'s valuation of attribute *k*. The term *ϵ*_ij_ captures unobserved taste variation across hospitals that is not quantified by hospital attributes ***x***_ij_. The collection $\left\{\epsilon_{ij},j\in J_{g\left(i\right)}\right\}$ is assumed to be i.i.d. EV (0, 1). Patient *i* chooses the hospital associated with the highest indirect conditional utility. Let *D*_ij_ = 1 if patient *i* is observed to choose alternative *j*, and *D*_ij_ = 0 otherwise. Then,

$$D_{ij}=1\;\Leftrightarrow \;U_{ij}=\max\left\{U_{in},n\in J_{g\left(i\right)}\right\}.$$

This model can be estimated by Maximum Simulated Likelihood (Hajivassiliou, 2000).

There are various hospital attributes that are relevant for the patient's valuation of a provider. These can be broadly partitioned into two sets. The first set comprises those attributes that are conveyed by the GP who makes the mandatory referral. Among these, there are notably quality related attributes—waiting time, readmission rates and attributes related to the provider's managerial performance (early FT status; specialist hospital; waiting time, to the extent that this is only revealed once the referral has been made). Regarding these, one would not expect significant variation in valuations, to the extent that GPs use the same information and criteria. The second set comprises those that patients can obtain themselves (distance, provider type) and where personal attitudes and circumstances impinge on the valuations. We endow these with random coefficients to allow for heterogeneity in preferences. In doing so, we allow patient characteristics to interact with these hospital attributes, and we also allow GP characteristics to do so—we consider that this is particularly important with regard to the ISP dummy, because GPs act as gatekeepers and thus may or may not provide awareness of, and access to, for‐profit provider options.

Hence, we include an ISP dummy among those attributes in ***x***_ij_ that carry a random coefficient, that is, $x_{ijk}=1_{\left\{j\in I_{g\left(i\right)}\right\}}$ and *σ*_*k*_ ≥ 0. Heterogeneity in sorting into ISPs then operates through the interactions of *x*_ijk_ with ***z***_*i*_. By controlling for *i*'s health and GP *g*(*i*)'s referral pattern among ***z***_*i*_, the model allows us to identify differential sorting, conditional on access and health, with respect to other patient sociodemographics, such as ethnicity and neigborhood deprivation. Our MMNL model also endows the hospital attributes distance and 30‐day emergency readmission ratio with random coefficients.^21^ Interactions with all continuous variables are constrained to be linear, with the exception of local area deprivation where we include separate terms for the most and least deprived half of the distribution.

We consider two definitions of choice sets: by distance (nearest 20 providers), and by GP referrals over previous 3 years.^22^

We employ our structural demand model not merely for a descriptive analysis, but also as a means to carry our some counterfactual analyses. It is therefore important to consider whether our model can identify structural effects. We discuss identification in Appendix A.

## 4 RESULTS

### 4.1 Baseline estimates

Table 3 provides headline results.^23^ Patients have a preference for shorter travel distances, shorter waiting times, and higher quality. We find that specialist hospitals are more likely to be chosen and ISPs less likely to be chosen. The random coefficient parameters indicate significant heterogeneity in valuations of distance and ISPs, but no unobserved variations in the emergency readmission rate. This finding might be explained by patients deferring to their GP with regard to quality assessments (Beckert, 2018; Dixon & Robertson, 2009; Monitor, 2015). In an incomplete information setting like the one considered here, quality is likely assessed via the patients' GPs who possess superior information. GPs, in turn, may have relatively homogeneous information on hospital quality and thus are unlikely to vary significantly in their quality assessments.

**TABLE 3:** Mixed logit results: Hospital attributes

	Distance choice set			GP choice set		
	Coeff	SE	*p*‐value	Coeff	SE	*p*‐value
Distance						
Mean	−0.0895	0.0079	0.000	−0.0765	0.0065	0.000
SD	0.1187	0.0017	0.000	−0.0669	0.0014	0.000
ISP						
Mean	−1.6935	0.2076	0.000	−1.5723	0.2081	0.000
SD	2.9803	0.0907	0.000	2.8348	0.0899	0.000
Emergency readmissions						
Mean	−1.3277	0.1249	0.000	−0.9999	0.132	0.000
SD	0.0404	0.0472	0.392	0.0852	0.1662	0.608
Attributes w/out RC						
Early Foundation trust	0.8323	0.1484	0.000	0.4033	0.1462	0.000
Waiting times (weeks)	−0.0811	0.0143	0.000	−0.0778	0.0146	0.000
Specialist orthopedic Hosp	1.4939	0.2044	0.000	3.0488	0.1713	0.000

Notes: The sample includes all patients who had an elective hip replacement in financial year 2012/2013 and chose one of the ten closest hospitals to the centroid of their LSOA. The model also includes interactions of all hospital characteristics in Table 3 and age, an ethnic minority dummy, dummies for moderate (Charlson index equal to unity) and severe (Charlson Index exceeding unity) comorbidities, and GP practice referral Herfindahl–Hirschman Index and ISP referral share over the last three years. Random coefficients are estimated at the patient level.

Abbreviations: ISP, Independent Sector Providers; LSOA, Lower Super Output Area; NHS, National Health Service; RC, random coefficient.

We summarize other noteworthy findings.^24^ We find that less deprived patients are more likely to be treated at an ISP. There are a number of ways one can think about such differences in ISP use by local area deprivation. They may be a consequence of where ISPs are located. In 2012/2013, patients in the richest quintile who had an ISP as the closest provider were about 50% more likely to choose an ISP than patients in the most deprived quintile (32% vs. 22%). But ISP location alone does not explain the impact of deprivation on the likelihood of being treated at an ISP. Practitioners have also argued that social and cultural barriers prevent poorer patients to consider for‐profit providers (or forego comprehensive healthcare altogether).

We also find that ethnic minority patients are less likely to be treated at an ISP. This can be explained by the distribution of ISPs across England with respect to ethnicity that follows a similar pattern as the distribution with respect to deprivation. In 2012/2013, 31.9% of white patients had an ISP as their nearest hospital, as did 25.1% of ethnic minority patients. But while 20.4% of white patients were treated by an ISP, only 8.2% of ethnic minority patients were. These statistics corroborate our estimation result and show that the difference between ISP use by ethnic minority status is therefore not just the result of the geographical distribution of providers.

Furthermore, we find that patients with underlying ill‐health are less likely to seek treatment at an ISP. This is expected as government regulation stipulates that patients must meet certain health criteria in order to be eligible for ISP treatment. Similarly, we find that ill‐health reduces the likelihood of treatment at specialist hospitals.

Our estimation results also show that patients who are registered with GP practices with high referral concentrations or low prior referral shares to ISPs are less likely to choose an ISP. This finding is consistent with an important role played by GPs in the decision making process. This finding suggests potential imperfections, or frictions, in the market for NHS‐funded care attributable to GP referral practice. Table 1 provides corroborating evidence. It highlights that there is a strong correlation between prior GP referral patterns and choosing an ISP. Comparing the concentration of GP practices referrals across patients in our sample shows that concentration does increase slightly with deprivation, with a mean of 0.62 for those in the most deprived fifth of areas, compared to 0.58 in the least deprived quintile.^25^ There is very little difference in mean concentration by ethnic minority status (0.6 for the white population and 0.59 for ethnic minorities).

In summary, our results show that ISP location, patient sociodemographic and health characteristics, as well as institutional frictions in the GP led referral practice contribute to patient sorting between public and private hospitals. Our counterfactual simulations, reported in Section 5 below, explore the relative importance for patient sorting of ISP geography vis‐à‐vis patient and GP referral characteristics.

### 4.2 Who benefits from choice enhancing reforms? And are any benefits spread equally?

Table 2 shows that for‐profit providers outperform public providers in terms of average waiting time and quality, measured in terms of readmission rates. This raises the question whether gains from getting access to for‐profit providers following the reforms benefit patients equally, and whether existing inequalities are attenuated or exacerbated. Our choice analysis permits some indicative assessment of these questions.

The well‐known conventional log‐sum, inclusive value expressions^26^ provide measures of patient utility from the underlying choice opportunities, that is, from the choice sets they have access to. Within our mixed logit framework, these measures can be constructed on the basis of estimates of the nonrandom coefficients, as an approximation to the analytically less tractable random coefficient counterparts. As we are only interested in their changes due to the reform, rather than in their absolute levels, between different patient groups we consider this approximation benign.^27^ We calculate these measures at the patient level for GP choice sets, with and without ISPs and conditional on ethnicity and deprivation quintiles. Tables 4 and 5 present quartiles of their respective distribution.

**TABLE 4:** The value of choice opportunities with and without ISPs, by ethnic minority status

	25th	km^a^	Median	km^a^	75th	km^a^	Interquartile range ^b^
All							
Majority	−1.582		−0.6166		0.059		1.641
Minority	−0.266		0.732		1.395		1.661
NHS							
Majority	−1.768		−0.736		−0.005		1.763
Minority	−0.29		0.717		1.39		1.680
Change (all—NHS)							
Majority	0.19	2.08	0.12	1.33	0.064	0.71	
Minority	0.024	0.18	0.015	0.42	0.005	0.04	

Notes: Simulations, based on the mean mixed multinomial logit coefficient estimates from the distance choice set model. ^a^ Reduction in average distance equivalent to utility gain, implied by mean distance coefficient estimated in distance choice model (−0.0895 for ethnic majority, and −0.1327 for ethnic minority). ^b^ Interquartile range.

Abbreviations: ISP, Independent Sector Providers; NHS, National Health Service.

**TABLE 5:** The value of choice opportunities with and without ISPs, by deprivation quintile

Deprivation quintile	25th	km^a^	Median	km^a^	75th	km^a^	Interquartile range^b^
All							
1	−1.702		−0.937		−0.316		1.387
2	−1.703		−0.823		−0.072		1.631
3	−1.921		−0.673		0.102		2.024
4	−1.171		−0.283		0.229		1.401
5 (most deprived)	−0.383		0.141		0.574		0.957
NHS							
1	−1.921		−1.106		−0.408		1.512
2	−1.901		−0.978		−0.160		1.741
3	−2.134		−0.800		0.038		2.172
4	−1.278		−0.355		0.193		1.471
5 (most deprived)	−0.451		0.088		0.552		1.003
Difference							
1	0.218	3.77	0.170	2.94	0.093	1.61	
2	0.198		0.155		0.087		
3	0.213		0.126		0.064		
4	0.107		0.072		0.036		
5 (most deprived)	0.068	0.59	0.053	0.46	0.022	0.19	

*Notes*: Simulations, based on the mean mixed multinomial logit coefficient estimates from the distance choice set model. ^a^ Reduction in average distance equivalent to utility gain, implied by mean distance coefficient estimated in distance choice model (−0.0578 for least deprived, and −0.1154 for most deprived patients). ^b^ Interquartile range.

Abbreviations: ISP, Independent Sector Providers; NHS, National Health Service.

The bottom panel of the Table 5 shows, across its columns, quartiles of the increase in the log‐sum expression due to the expansion of choice opportunities, by deprivation quintile across its rows. It shows that the top three quintiles of the deprivation distribution, quintiles 1–3, that is, the richest 60% of patients, benefit most from being given access to ISPs—the lower quartiles of their log‐sum expressions increase on the order of 0.2, the upper quartiles between 0.06 and 0.09—, about twice as much as the fourth—with lower quartile of 0.1 and upper quartile of 0.03—and about three times as much as the bottom quintile—with lower quartile of 0.07 and upper quartile of 0.02. Similarly, ethnic majority patients benefit considerably more, about six times as much as minority patients.

A measure of the inequality in choice opportunities is the interquartile range (IQR) of the respective inclusive value distribution. Comparing the IQR for GP choice sets with and without ISPs shows that patients from the upper three deprivation quintiles experience a reduction in inequality in choice opportunities as a consequence of the reform, while the bottom two quintiles do not. Similarly, the inequality in choice opportunities for ethnic majority patients is attenuated post reform, while it is practically unchanged for ethnic minority patients.

Tables 4 and 5 also present the average reduction in distance to any hospital in the choice set that would be equivalent to the gain experienced by the respective stratum of the patient population. These distance reductions are implied by the estimated distance coefficients from our choice model which allows for distance effects that are heterogeneous across ethnicity and deprivation.^28^ Such heterogeneity is more pronounced with regard to deprivation than ethnicity, and hence while, for example, for the median patient the 10 percentage point utility difference between ethnic majority and minority translates into roughly a threefold difference in the average distance effect (1.33 vs. 0.43 km), the comparable 11 percentage point utility difference between the least and most deprived patients translates into a more than sixfold distance effect (2.94 vs. 0.46 km).

These findings to some extent can be explained by hospital geography. The very modest benefit accruing to, and the largely absent inequality attenuation for, ethnic minority patients at least in part is attributable to minority patients predominantly living in big cities, esp. London, where few ISPs are located. Similarly, the more pronounced benefits of the reforms accruing to patients living in rich areas is likely due to ISPs being located in wealthier parts of the country, outside London.

## 5 COUNTERFACTUAL SIMULATIONS

We now present results of a series of counterfactual simulations. The simulations aim at exploring the relative contributions of potentially important drivers of patient choice that are unrelated to hospital attributes and instead derive from patient characteristics and guidance that patients receive from their GP. Specifically, with regard to access, we ask what would the relative benefits between rich and poor, and ethnic majority and minority, patients be but for differences in patient health, preferences, GP referral practice, and health geography. With regard to cost, we ask whether a level playing field exists between public and for‐profit providers, or whether differential access entails adverse consequence for the allocation of costs that disadvantages public hospitals.

We emphasize that we do not interpret the results presented here as predictions from policy simulations but rather as stress tests of the model, in order to assess the relative substantive importance of the respective drivers of choice. In Appendix B, we discuss details on the underlying assumptions and caveats of our simulations.

### 5.1 Mechanisms

Table 6 shows the predicted ISP shares by income quintile and ethnic minority status when we successively shut down mechanisms that could contribute to the differences in ISP use.

**TABLE 6:** Simulation results: Expected ISP volumes by deprivation quintile and ethnicity

	Data			Simulations				
	Patients	Share ISP	Model	a) Health	b) Ethnicity	c) Prefs	d) GP effects	e) DCS
Deprivation								
1 (least)	15,176	0.222	0.264	0.299	0.298	0.298	0.305	0.422
2	15,106	0.217	0.260	0.293	0.293	0.295	0.301	0.420
3	13,830	0.205	0.251	0.284	0.284	0.287	0.293	0.416
4	10,634	0.162	0.227	0.265	0.264	0.269	0.276	0.402
5 (most)	7949	0.119	0.207	0.252	0.251	0.257	0.263	0.387
Ethnicity								
Majority	60,573	0.198	0.250	0.284		0.287	0.293	0.414
Minority	2122	0.075	0.154	0.242		0.244	0.229	0.372

The first two columns give the total number of hip replacements and the share of hip replacements conducted by ISPs by the local area deprivation quintile of the patient's lower super output area. The third column gives the mean predicted probability that a patient chooses an ISP using the GP choice set model. These predicted probabilities are calculated by summing the predicted probabilities for ISP alternatives for each patient. The final five columns consider successive, cumulative simulations. The fourth column gives all patients the mean underlying health, column 5 equalizes ethnicity, column 6 equalizes preferences by removing interaction between deprivation and hospital attributes (top panel) and ethnic minority status and hospital attributes (bottom panel).

Abbreviations: DCS, distance choice set; ISP, Independent Sector Providers.

In step 1, we equalize health across the socioeconomic distribution by giving all individuals the mean health across all our health measures. The gradient in ISP use between richest and poorest patients shrinks from 5.7 percentage points (26.4%—20.7%) to 4.7 percentage points (29.9%—25.2%), This equates to an absolute decrease of 1 percentage point, which is 17.5% of the original gradient. Step 2 equalizes ethnicity, all patients are assumed to behave like those who are White British. This has minimal effects on the distribution of ISPs by deprivation, as there are only a small number of ethnic minority patients. In Step 3, we equalize preferences by eliminating interactions between hospital attributes and deprivation. Again, there is very little change in the predicted number of ISP patients and the deprivation gradient. Simulation step 4 removes all interactions with GP characteristics. This results in a small increase in ISP use for all income quintiles, on the order of 2% for all income quintiles. The final step replaces the GP choice set with the distance choice set, and assumes that all patients choose between the 20 closest providers. This has a sizable impact on the share of patients treated by ISPs in all income quintiles, as the distance choice set is typically larger than the GP choice set, particularly in terms of ISPs. The gradient in ISP use between richest and poorest patients shrinks again, to 3.5 percentage points (42.2%–38.7%). As health, ethnicity and GP referrals have been equalized, the only remaining differences come result from hospital geography. This remaining 3.5 percentage points, or 61% of the original difference from the model is therefore attributable to differences in the geographic distribution of hospitals.

A similar set of results are observed when we consider the same set of simulations by ethnicity. The model predicts a gap of 9.6 percentage points in ISP use between white and ethnic minority patients (25% vs. 15.4%). Step 1, which equalizes health, reduces the difference in ISP use by ethnicity from 9.6 percentage points to 4.2 percentage points, or 56% of the original difference. Step 2 and 3 are equivalent in this simulation and have almost no impact on ISP use. Step 4, which removes GP preferences, reduces ISP use by minorities. As for the income simulations, Step 5 also has a large impact on predicted ISP use. For white patients, applying the distance choice set increases ISP use by 41% compared to 63% for ethnic minority patients. After step 4, the predicted difference in ISP use remains 4.2 percentage points or 44% of the total.

It may be worth noting that any ethnic minority effect is likely to be accentuated by London. There are very few ISPs in London, but a high share of ethnic minority patients. In the raw data, the share of ethnic minorities that use ISPs is 7.7% when London is included, and 10.8% when London is excluded. ^29^

Taken together, the simulations reveal three points of note. First, differences in health do explain a sizable and important portion of the gradient in ISP use by local area deprivation and ethnicity. Second, patients in poorer areas are presented with a narrower range of hospitals when making choices, even when taking the local health economy into account. Third, our simulations show that even removing differences in health, preferences, and the GP's impact on the set of providers offered, ethnic minority patients and those living in more deprived areas are less likely to choose an ISP. This can only be attributed to the geographical location of ISPs. So location matters. Conditional on hospital location, this response from patients may well be efficient. The question in policy terms is whether such a geographic distribution of service provision is desirable.

### 5.2 The costs of sorting

Our parameter estimates and simulations illustrate that differences in underlying health are important sources of sorting. This may represent an efficient allocation of resources as ISPs do not have the specialist facilities needed to appropriately treat those with complex needs. However, the payments the NHS makes to providers have only very limited gradations in payments to take account of differences in patient health that affect the costs of treatments patients receive. Table 7, and Appendix C, provides an overview of national tariffs for the HRGs in our sample and their distribution.

**TABLE 7:** NHS tariffs and their distribution

HRG Groups	Total Patients	Tariff, £($)	Share prev em admits	Share ISP
HB12C: Major hip procedures for non‐trauma category 1 without CC	47,280	5382 (6728)	0.170	0.222
HB12A: Major hip procedures for non‐trauma category 1 with major CC	3100	8305 (10,381)	0.345	0.072
HB12B: Major hip procedures for non‐trauma category 1 with CC	3187	6021 (7526)	0.340	0.091
HB11C: Major hip procedures for non‐trauma category 2 without CC	1491	6579 (8224)	0.370	0.190
Other	7654	‐	0.324	0.089
Total	62,712		0.211	0.191

Abbreviations: HRG, health resource group; ISP, Independent Sector Providers.

Source: NHS England 2012–2013 tariff information spreadsheet.

In this section we consider the implications for NHS hospitals and for competition when sorting occurs within HRGs. Specifically, we focus on the implications of patient sorting on hospital costs. Costs are an important driver of competition and typically taken as exogenous. But as we show, costs themselves can also be affected by changes to the competitive landscape, such as a the choice reforms considered in our study.^30^

Our data do not allow us to calculate the precise cost to the hospital of treating a particular patient. We therefore adopt a simplified approach where we assume that differences in cost are proxied for by length of stay. Estimates of costs per bed day vary, but we use the per day long stay payment in 2012/2013 of £231 per day (NHS England, 2013).

Restricting our attention to just those patients who have a “simple” HRG (HB12C), the data show that patients treated by NHS hospitals have a mean stay of 4.5 days, compared to 3.7 days for patient treated by ISPs. This shorter length of stay for ISP patients may be explained by two factors. First, ISPs may deliver care faster and discharge patients earlier. Second, patients treated by ISPs may be healthier and less costly to treat.

To investigate the importance of the latter factor, we run a linear model of length of stay for patients treated by NHS hospitals on a quadratic in age, sex, previous admissions and Charlson index, ethnic minority status and local area deprivation. We then use this model to predict the length of stay for all patients, given provider type. The mean is 4.44 days for NHS patients, and 4.26 days for patients treated by ISPs. This shows that the differences in the raw data are primarily driven by ISPs discharging patients sooner, but that patients treated by ISPs are also slightly less complex ^31^

Using these estimates, we conduct a counterfactual calculation where the 10,728 ISP patients replace a random sample of the same number of patient treated in an NHS hospital. To do this we use the sum of the predicted lengths of stay across all ISP patients, giving a total number of bed days of 45,653 days. We then draw a random sample of 10,728 patients from NHS hospitals from within the same HRG, and again calculate the predicted number of bed days. For the sample we draw, this is equal to 47,499 patient days. Their difference gives the total number of additional patient days at NHS hospitals if ISP patients had been treated by NHS hospitals, which is equal to 1846 patient days across the NHS or 14 days per year per NHS Acute Trust. Applying the cost per bed day of £231, this gives a total cost of £426,426 ($625,000). This is equivalent to the cost of an additional 77 hip replacements or 0.1% of the total number of hip replacements that took place in 2012/2013.

In summary, these calculations suggest that the limited gradation in HRG payments means that NHS hospitals and ISPs receive the same payment for treating “simple” patients, but that ISPs face lower patient contingent per treatment costs. This would imply that ISPs have higher profit margins, which are not due to ISPs being intrinsically more efficient, and that as a consequence of sorting ISPs receive an implicit subsidy from the taxpayer. This subsidy is quite small in magnitude. However, it is likely that there is a similar pattern in other procedures provided by ISPs such as knee replacements and cataract operations.

On the other hand, patient sorting may entail allocation and efficiency benefits elsewhere in the wider health care system: It may free up capacity at public providers to treat more complex patients faster, accelerate the formation of centers of excellence for certain procedures, and for suitable procedures and cases insert the publicly funded option as direct competitor to private insurance, thereby invigorating competition in the private health care market. Quantifying such potential benefits goes beyond the scope of this study.

Any patient sorting that is correlated with treatment costs will have similar implications in all DRG systems. These costs must be taken into account when regulating healthcare markets or designing policy, and must be weighed against the advantages of a limited number of DRG codes. One possible policy response would be to offer lower payments to ISPs or an additional low risk band. However, there may be a concern that this would reduce the willingness of ISPs to treat NHS‐funded patients.

## 6 CONCLUSIONS

In this paper we investigate effects of public versus for‐profit provision of care made possible by reforms in the English NHS that enabled privately owned for‐profit surgical centers to access the market for NHS‐funded hip replacements. We show that poor and ethnic minority patients benefitted less from these new providers than wealthier and white patients, and that the reforms attenuated existing inequalities in choice opportunities and access for the latter patient groups, while doing nothing to inequalities of the former. We identify patient health and the geographic distribution of for‐profit and public providers as dominant drivers of differential benefits, with primary care referral practice playing a secondary role. Finally, while acknowledging possible allocation and efficiency benefits of patient sorting whose quantification goes beyond this study, we estimate that under the current capitated reimbursement NHS funding architecture patient sorting can induce non‐negligible costs to providers. To the extent that the composition of patient risks differs between private and public providers, such costs may put public providers at a competitive disadvantage.

Our findings have several important implications for policy, both within the English NHS and in other country and policy settings. First, eligibility criteria and the geographic distribution of providers have important implications for how consumers sort across providers. In our context, we show how health criteria and the opening up of pre‐existing private hospitals to public patients led to ISPs treating a higher share of rich and white patients. Similarly, reforms that encouraged entry of private primary care provision in Sweden disproportionately benefitted patients who lived in urban areas and those above median incomes (Anell, 2015); Suskind et al. (2015) raise similar concerns with regard to ASCs in the US. This type of sorting is not necessarily inefficient when the set of providers and eligibility criteria is taken as given, but may still be of concern to policy makers.

Second, our results support existing work that highlights ethnic disparities in health care access (Cookson et al., 2016; Dixon et al., 2010; Fiscella et al., 2000; Nelson, 2002). Our results demonstrate that existing inequalities in health care access were only partly attenuated by the reforms. Surveys of GPs and studies of differential take‐up of screening suggest that there are likely to be further barriers (Fisher, Audrey, Mytton, Hickman, & Trotter, 2014; Moser, Patnick, & Beral, 2009). This may be regarded as undesirable by politicians and the public, in a health care system that strives for the provision of equal treatment for equal need.

Our work also highlights the implications for the re‐allocation of costs of allowing for‐profit providers to compete for the market of publicly funded services. In many health care systems, payments are fixed or have limited differentiation, therefore new providers that advantageously select risks limit the incumbents' ability to cross‐subsidize. Our results illustrate this point. Such concerns are particularly relevant at present, when post‐Great Recession austerity means that budgets for the NHS and other public services are particularly tight. Our results also compound concerns about budget re‐allocation effects that have been raised in the case of recent US Veteran Affairs proposals.^32^

Furthermore, our analysis contributes to the emerging discussion of provider governance in publicly funded services when the role of the State as service provider recedes and is transformed to market design and the oversight of public–private competition. As regards healthcare systems, this discussion is ongoing in many jurisdictions. Saltman and Duran (2016) and Leichsenring, Rodrigues, Winkelmann, and Falk (2015), for example, highlight the complexities of changes in healthcare governance with examples in Germany, Spain and Sweden.

Our analysis emphasizes that allowing private providers to enter the funded elective care market expanded the set of notional choice options. Our results show that, ceteris paribus, actual gains from this expansion primarily accrue to those patients who are best placed to exercise choice. This may be as a consequence of where they live, their own innate ability or sociodemographic characteristics, or due to other aspects of the choice process such as the importance of advice from GPs.

There may be some gains for all patients, if the increase in competition from private providers drives improvements in quality, which we do not examine in this paper. However, existing work on this reform indicates that while there was some improvement in efficiency of public hospitals (Cooper et al., 2018), there is no evidence of quality improvements (Kelly & Stoye, 2020).

Our work suggests avenues for future research. If choice is to be the driving force behind competition and a driver of quality improvement, more work is needed to understand information imperfections and other frictions that may limit choice for all, and for some groups in particular.

## CONFLICT OF INTEREST

Authors are responsible for disclosing all financial and personal relationships between themselves and others that might bias their work.

## Data Availability

The data that support the findings of this study are available from National Health Service (NHS) Digital. Restrictions apply to the availability of these data, which were used under license for this study.

## ENDNOTE

^1^ Critics of the Veteran Affairs proposal warn that many hospitals of the 153‐year old system may starve and close (New York Times, 12 January 2019).

^2^ Gaynor et al. (2016) consider Coronary Artery Bypass Graft surgery and this is not a market where Independent Sector Providers (ISPs) operate.

^3^ See, for example, Fiscella et al. (2000), p. 2580.

^4^ Our counterfactuals go beyond the finding that utilization declines with distance (e.g., Wang, 2012).

^5^ Wave 1 Independent Sector Treatment Centers (ISTCs) had block contracts, while Wave 2 ISTCs no longer did.

^6^ See, for example, the CMA's Final Report of the Private Healthcare Market Investigation (2014), chapters 4 and 6, available at https://www.gov.uk/cma-cases/private-healthcare-market-investigation. We recognize, however, that for‐profit providers eventually may also enter less affluent areas, with a view to benefit from the reforms and treat National Health Service (NHS) funded patients alongside others, so that the inequalities shown in our analysis may be abated.

^7^ Our data, however, do not permit to identify whether a patient is eligible to be treated by a for‐profit provider. Not only does this depend on the patient's clinically relevant characteristics, but also on the case assessment by the referring GP and the assessment (anamnesis) by any for‐profit provider. Our approach therefore attempts to control for patient characteristics that are relevant for the likelihood of the patient being able to choose a for‐profit provider.

^8^ For the purpose of our analyses, we do not distinguish between ISTCs and private hospitals. We carried out a number of robustness checks to examine whether the gradient of choice of ISTCs with respect to deprivation differs from that of private hospital. It does not. This is evidence against a conjecture that block contracts for Wave 1 ISTCs may generate the deprivation effects our empirical analysis identifies. The robustness checks are available on request.

^9^ These shares are much lower than the share of people of ethnic minority patients in the population, due to the age structure of the ethnic minority population in England

^10^ It is calculated using the comorbidities recorded at the point of the hip replacement admission. The Charlson Index predicts ten‐year mortality using 22 comorbidity conditions. Each condition is scored a 1, 2, 3, or 6, depending on the severity of the condition, and is calculated on the basis of all diagnoses recorded in hip replacement admission. See Sundararajan et al. (2004) for more details on the Charlson Index. We group patients into three categories: a score of zero for no comorbidities; a score of 1 for “moderate comorbidities”; and a score of more than one for “severe comorbidities.”

^11^ Comparing these measures with the reported underlying health recorded for the 60% of the sample that responded the patient recorded outcome measures (PROMs) survey illustrates that the health measures we use pick up different elements of ill health. Of those that report ever having cancer in PROMs, 79% have had an elective admission to hospital over the previous 3 years, compared to 53% for all other patients, emergency admissions were 10 percentage points higher (29% verses 19%), and cancer patients were twice as likely to have a Charlson index score of 2 or more (15% verses 7%). By contrast, for those reporting high blood pressure, the shares with prior emergency and elective admissions are both only 2 percentage points higher than the rest of the sample, whereas the share of those with a Charlson Index of 2 or more is 6 percentage points higher.

^12^ This is also true when we use the more detailed PROMs questionnaire available for two thirds of the sample. Even for those who report having a previous stroke or heart attack, 10% have a hip replacement conducted by an ISP.

^13^ Lower Super‐Output Areas are statistical geographical aggregation units with no administrative jurisdiction, similar to a census tract, and are designed to be as homogeneous as possible with respect to population composition. They contain an average of 1500 individuals. There are approximately 32,500 LSOAs in England.

^14^ The Index of Multiple Deprivation is a local area based measure of deprivation produced by the UK government that includes measures of income, employment, health deprivation and disability, education skills and training, barriers to housing and services, crime and the living environment. We use the version produced in 2010. Please see https://www.gov.uk/government/statistics/english-indices-of-deprivation-2010 for more details. This is the same measure used to capture the economic conditions as Gaynor et al. (2016).

^15^ This is given by the sum of squared referral shares of each hospital that the GP practice refers to.

^16^ For example, PROMS are more complete for NHS hospitals than ISPs, and there is a concern that missing data could be nonrandom. We therefore decided not to construct quality measures from PROMS data.

^17^ Moscelli, Gravelle, Siciliani, and Gutacker (2018) argue that unobserved selection may induce bias in the regression we use to predict readmissions, so that our quality measure might be contaminated. We allow for this in our demand model by interacting the readmission rate measure with, among others, the GP level ISP referral share over the last three years. We are grateful for a commentator to suggest this approach.

^18^ See footnote 41 in Gaynor et al. (2016).

^19^ In line with the informational content of our data, which identify a patient's GP practice, but not the individual GP, in much of our discussion we refer to GP and GP practice synonymously.

^20^ We consider that hospital fixed effects remove potential endogeneity of waiting times. Nonetheless, as in Beckert (2018), we also implemented a control function approach (Blundell & Powell, 2004) where we instrumented waiting times 2012–2013 with waiting times 2011–2012. We found that the resulting estimated coefficients, notably on waiting times, are not statistically different from the ones we report below. First‐stage and control function results are available from the authors on request.

^21^ As expected, the variation in the coefficient on the latter turns out not to be statistically significant.

^22^ We acknowledge that our approach implicitly assumes that GPs do not react to new information, notably on provider quality, or alter the weight they place on provider attributes. Moscelli et al. (2016) show that the average waiting time and quality of hip replacements have changed between 2002/2003 and 2012/2013; average waiting time has fallen monotonically, but measures of average quality have only fallen from around 2006. So it is not inconceivable that GPs might adapt the choice sets they offer to patients over time.

^23^ Due to the interactions of hospital attributes and patient characteristics and the random coefficients, the full model has 70 estimable parameters. Space restrictions do not permit us to present all estimates in the paper. The remaining parameter estimates are available on request.

^24^ These relate to various interactions of patient characteristics and hospital attributes whose estimates are available from the authors.

^25^ The exception to this pattern is London, where concentration decreases with local area deprivation

^26^ Conditional on $\beta_{i}={\left[\beta_{ik}\right]}_{k=1,\ldots ,K}$, the inclusive value of the choice set $J_{g\left(i\right)}$ is $E\left[\max_{j\in J_{g\left(i\right)}}U_{ij}|\beta_{i}\right]=\ln\left(\sum_{j\in J_{g\left(i\right)}}\exp\left(x_{ij}^{\prime }\beta_{i}\right)\right)$. See Williams (1977); Small and Rosen (1981) for a derivation of the log‐sum formula, and McFadden (1981), Section 5.8, for a more general discussion of social surplus functions in the context of probabilistic choice. When basing our analysis on the nonrandom coefficients only, we consider that the results are, as always, conditional on just identifying assumptions, which include that the specification of conditional indirect utility, with all its interactions among the regressors, is sufficiently rich to capture preference heterogeneity to such an extent that the random terms are conditionally uncorrelated with the regressors.

^27^ We effectively compare differences across patient groups. This is like a Difference–in–Difference approach: For each patient group, this differences out the base alternative; we then compare these differences across groups.

^28^ These distances can be calculated as difference in log‐sum (or inclusive value) expressions, divided by the estimate of the respective coefficient on distance.

^29^ The same is not true for deprivation: The share of ISP use with and without London is very similar.

^30^ There exists a large theoretical and empirical literature on the effects of choice on other dimensions of competition. For example, the literature of the effects of competition on quality is surveyed by Gravelle, Santos, Siciliani, and Goudie (2012). The theoretical literature (Brekke et al., 2006, 2011; Hehenkamp & Kaarbøe, 2020; Herr, 2011) often focusses on optimal location choice. Our analysis takes locations as exogenous (see discussion in Section 2.1). The empirical literature finds both, positive effects (Cooper et al., 2018; Gaynor et al., 2016; Kessler & McClellan, 2000), and negative effects (Gowrisankaran & Town, 2003). We take any potential benefits, for example, as identified for example, by Cooper et al. (2018), as given.

^31^ We check the robustness of this result by also running a model that includes the comorbidities recorded in PROMS data, which are not present in Health Episode Statistics. The results do not change. Our results are consistent with (Siciliani, Sivey, & Street, 2013), who find that average lengths of stay were shorter in public and private treatment centers than NHS hospitals, and that these differences persisted after controlling for differences in age, diagnoses, deprivation and region.

^32^ New York Times, 12 January 2019.

## APPENDIX A Identification

### A.1

We employ our structural demand model not merely for a descriptive analysis, but also as a means to carry our some counterfactual analyses. It is therefore important to consider whether our model can identify structural effects. A threat to identification arises if the error of the indirect utility model were correlated with included regressors, that is, if some regressors were endogenous. The primary concern thereby relates to the possible endogeneity of hospital waiting times and quality. Such concerns have been considered and discussed in similar studies of hospital choice.

Beckert (2018) uses the control function approach (e.g., Heckman and Robb (1985); Blundell and Powell (2003); Petrin and Train (2010)) to guard against endogeneity of waiting times. Gaynor et al. (2016) suggest including hospital fixed effects to mitigate concerns about the endogeneity of waiting times. These fixed effects capture time, patient and GP invariant unobserved attributes such as reputation and what Gaynor et al. refer to as past experience with the hospital. Gutacker et al. (2016) control for such past experience directly, by including lagged instead of contemporaneous waiting times.

Gaynor et al. (2016) point out that quality, as waiting time, responds to demand and thus is endogenous, essentially as a learning‐by‐doing or expertise effect; see also Birkmeyer et al. (2002); Silber et al. (2010); Halm, Lee, and Chassin (2002). Their study again refers to hospital fixed effects as a means to address the endogeneity of quality. Gutacker et al. (2016) point to unobserved patient selection, either on the part of patients themselves or on the part of the provider, as another possible, albeit unlikely, source of endogeneity of quality.

There are three dimensions of variation in the data: within hospital site across GPs; within GP across patients; and across hospital sites. We follow Gaynor et al. (2016) by including hospital site fixed effects in our mixed logit model. They can be identified and estimated through the within‐site variation across GPs and patients. Moon, Shum, and Weidner (2018) discuss the use of fixed effects in such models to accommodate endogeneity concerns. ^33^ Berry (1994); Berry, Levinsohn, and Pakes (1995) use instruments to deal with endogeneity in cross‐sectional data settings. Since our data have a panel structure, we are able to estimate hospital site level unobservables to the extent that they do not vary across patient within GP practice. The within‐variation also identifies the effects of hospital attributes interacted with patient and GP characteristics. The between‐hospital variation identifies the effects of hospital attributes on hospital market shares. ^34^ One way to think about this latter point is that, in a second stage estimation, the hospital fixed effects can be regressed on the hospital attributes.

Furthermore, our quality measure, readmissions rates at the hospital site level, is a case adjusted measure, that is, it sets observed readmissions in relation to predicted admissions and thus captures that quality element that is orthogonal to what the hospital's patient profile would predict. It therefore is exogenous by construction. Our construction of this measure is analogous to the construction of standardized or case adjusted mortality rates published by the Department of Health (e.g., Summary Hospital Mortality Index, SHMI). ^35^ Summary Hospital Mortality Indexs are only published by NHS Digital for public hospitals, at the trust level.

We posit that this quality measure is in the GP's information set and as such a hospital attribute that flows into the mandatory advice that the GP has to give to the patient at the point of patient choice and referral. We believe it is superior to other possible quality measures, such as patient reported outcome measures (PROMS) which suffer from self‐reporting bias and, in any event, are not consistently recorded for ISPs so that we cannot use them due to incomplete coverage. A possibly relevant additional source of information on quality might be the performance information from the National Joint Registry on the orthopedic surgeon to whom the referral to a hospital would lead. In our data, we neither observe the surgeon responsible for the actual procedure nor the surgeons that would be possible alternatives at other hospitals in the patient's choice set. So we are unable to incorporate surgeon information among the hospital attributes in our model. We also note that some surgeons may work across hospital sites, which again we would not observe in the data and hence we could not account for.

## APPENDIX B Simulations: Underlying Assumptions

### B.1

In the interest of transparency, even at the risk of appearing overly apologetic, we start with a detailed discussion of caveats to our simulation analysis. The main reason why we caution against over‐interpreting the level predictions of the counterfactual simulations is that they are likely to be contaminated by several sources of potential bias. In general, our maximum likelihood (ML) estimation methodology assesses goodness of fit in terms of the value of the likelihood function at the ML estimates, not in terms of a criterion based on prediction errors.

That aside, discrete choice models like the ones estimated here assign nonzero probability to every choice alternative in the choice set. In our context, this is relatively unproblematic as far as NHS hospitals are concerned, but potentially problematic when considering Independent Sector Providers (ISPs) because some patients may not be considered eligible for treatment by ISPs, due to health conditions unobserved by the econometrician. Nonzero predicted choice probabilities for ISPs induce an upward bias in counterfactual predictions of ISP choice.

Moreover, when predicting ISP choice conditional on a specific patient characteristic, for example, then the formally correct approach to calculate the predicted conditional choice probability is to integrate out all other patient characteristics, conditional on the specific characteristic under consideration. But the multivariate conditional distribution of such other characteristics is difficult to estimate, even with the rich data we have, and attempts to estimate it entail risks of bias and nonrobustness of their own.

A straightforward way to estimate predicted ISP uptake, conditional on a patient characteristic, say deprivation, is to sum the predicted choice probabilities conditional on that characteristic. If deprived patients have relatively bad health and hence their predicted ISP choice probabilities are relatively low, then in the formally correct approach these low predicted probabilities would have higher weight in the calculation of expected ISP choice. Hence, simply summing unweighted predicted ISP choice probabilities will add to the aforementioned upward bias. Conversely, if wealthy patients are in relatively good health and hence their predicted ISP choice probabilities are relatively high, then these high predicted probabilities would have higher weight in the correct approach to calculation of expected ISP choice, and the sum of unweighted predicted ISP probabilities will tend to underestimate the expected ISP uptake. Therefore, comparing predicted ISP uptake by sums of predicted choice probabilities for high and low deprivation patients will tend to underestimate the discrepancy between these patient groups. Acknowledging this limitation of our approach to prediction, we accordingly limit our interpretation to the comparison of proportionate changes in ISP uptake in a number of counterfactual simulations.

We also acknowledge that, when presenting our simulations, we ignore the additional variability induced by using parameter estimates. This biases standard errors downwards and makes our predictions, notably the predicted reductions in the gradients in simulated volumes, look more precise than they truly are.

Our assumptions for these simulations are as follows. When considering counterfactual expansions in choice sets, we assume that the costs to the GP of providing wider choice are minimal, that is, there is no GP‐level capacity constraint. Furthermore, we assume that there is no capacity constraint at the hospital level, so that additional patients treated at a hospital under the counterfactual do not change the attributes of that hospital. Endogenising capacity and other supply‐side responses would require a general equilibrium model which is beyond the scope of this paper. Given that hospital attributes such as waiting times may change, the predicted demand shift is an upper bound of the expected effects. ^36^ Hospitals that are predicted to face increased patient demand under the counterfactual with given attributes are likely to increase waiting times if they are capacity constrained, which in equilibrium would dampen predicted demand. Conversely, hospitals that are predicted to face diminished demand under the counterfactual with given attributes are likely to be able to reduce waiting times, which in turn would enhance predicted demand. Beckert and Schiraldi (2017) provide an equilibrium analysis in which waiting times respond endogenously to changes in demand.

## APPENDIX C APPENDIX C: NHS Tariffs

### C.1

Table 7 shows the main NHS tariffs or payments that are applied to hip replacement patients in our sample. These tariffs are set to reflect the average cost of providing a particular bundle of care. The first and second column show the number of patients in each health resource group (HRG) category and the associated tariff. Three‐quarters of the sample fall into a single category (Major Hip Procedures for non‐Trauma Category 1 without Complications or Comorbidities HB12C), which had a price of £5832 in 2013/2013 (NHS England, 2013). Only around 10% fall into HRGs for those with complications and comorbidities, which are associated with higher tariffs.

The third column of Table 7 shows the share of patients with previous emergency admissions to hospitals. There are two points to note here. The first is that, as might be expected, the share of patients with previous emergency admissions for the “simple” tariff is approximately half (0.17) that of patients for tariffs with some and major complications (0.345 and 0.34 respectively). Second, although the share of patients with previous admissions is lower for those in the “simple” HRG, there is a considerable share that do have underlying health conditions. In addition to the 17% who have had a previous emergency admission, 53% have had a previous elective admission, 27% have a nonzero Charlson Index, and 10% are over 80. This suggests that the true costs to hospitals of treating patients within the same HRG are likely to vary. Any sorting according to underlying health and the associated costs would not be reflected in tariff payments. In particular, if healthier patients choose ISPs, the payments received by NHS hospitals and ISPs will be the same, but the average cost per patient treated by an NHS hospital will rise. This limits the ability of NHS hospitals to cross‐subsidize across patients.

The fourth column of Table 7 shows the share of patients within each tariff that are treated by ISPs. ISPs treat 22% of patients in the “simple” HRG, but only 9.1% of those within the tariff for those with “complications and comorbidities” and 7.6% within the tariff for those with “major complications and comorbidities”. This sorting across tariffs is compatible with the goals of the ISP reforms, as competition even within a segment of the market may be sufficient to generate pressure to improve quality for all. The distribution is also consistent with improving patient welfare, as NHS hospitals are likely to be better equipped to deal with patients with worse underlying health. NHS hospitals may see a higher share of complicated patients, but they are compensated for the associated costs by higher tariff payments.
